# High Clinical and Genetic Similarity Between Chronic Pancreatitis Associated With Light-to-Moderate Alcohol Consumption and Classical Alcoholic Chronic Pancreatitis

**DOI:** 10.1016/j.gastha.2022.11.012

**Published:** 2022-12-08

**Authors:** David C. Whitcomb

**Affiliations:** Division of Gastroenterology, Hepatology and Nutrition, Department of Medicine and Departments of Cell Biology & Molecular Physiology, and Human Genetics, University of Pittsburgh and UPMC, Pittsburgh, Pennsylvania, USA

## Rethinking Alcohol-Associated Pancreatitis

Relationships between alcohol and chronic pancreatitis are complex. Most studies of pancreatitis coming out of Europe and South Africa in the 1960–2000 time period reported that severe alcohol abuse was responsible for greater than 90% of cases of chronic pancreatitis.^1^ However, a few studies from the United States and other parts of Europe suggested that the rate of alcohol pancreatitis was lower than 90%, (eg about 50%)^1^ and that other factors such as hereditary pancreatitis genes could also cause chronic pancreatitis. While much progress has been made on understanding non-alcohol-associated pancreatitis, the study by Wang et al^2^ provides paradigm-shifting insights into why some alcohol drinkers get chronic pancreatitis.

Alcohol by itself does not cause chronic pancreatitis. For example, the clinical evaluation of 100 patients in a VA alcohol rehabilitation unit only identified chronic pancreatitis in 3% of very high-risk subjects.^3^ Animal studies also suggest that even high-dose alcohol consumption does not cause chronic pancreatitis alone and may actually reduce inflammation.^4^ In alcohol-fed animals, pancreatitis must be triggered by another mechanism, and then the alcohol-related pancreatitis processes can be studied.^4^

The first (sentinel) acute pancreatitis event (SAPE) markedly changes the pancreas and makes it hypersensitive to subsequent injury or stress.^1^^,^^5^ This fact is recognized in clinical practice as patients with acute pancreatitis are evaluated aggressively for gallstone disease and undergo cholecystectomy with any signs of biliary stones or sludge.^1^ If no other major non-alcohol etiology is found, these patients with “idiopathic” pancreatitis may also be sent for cholecystectomy to prevent recurrence.^6^ For alcohol-associated acute pancreatitis, the only recommendation is to stop drinking (and stop smoking).

What triggers the first episode of acute pancreatitis in an alcohol-drinking individual? We know that trypsin activation inside the pancreas is a key step in triggering acute pancreatitis.^7^ Susceptibility to acute pancreatitis is related to the balance between trypsin activating conditions (eg hyperstimulation with increased intracellular calcium levels, *PRSS1* gain-of-function mutations) and protective mechanisms (eg high ductal pH, duct clearance, trypsin inhibitors including *SPINK1, CTRC,* and *PRSS3*), clinical acute pancreatitis only occurs when activation factors >> protective mechanism. The threshold stimulus to cause acute pancreatitis in an individual person depends on their innate (genetic) characteristics, their acquired biological state and a stochastic injury or stress triggering factor. Alcohol decreases the protective mechanism in acinar cells through multiple mechanisms, and these effects are dose-dependent.^8^^,^^9^ This may explain why, from a population standpoint, there is an increasing rate of acute and chronic pancreatitis with increasing alcohol consumption—with a minimum threshold of about 4-5 drinks per day.^9^^,^^10^

Why is there such heterogeneity in the risk and severity of acute and chronic pancreatitis among alcohol drinkers at mild-moderate and heavy-very heavy alcohol consumption? The first part of the answer depends on whether or not the patients continue drinking (and smoking) after their SAPE.^11^ Continued alcohol not only accelerates the transition from acute pancreatitis to chronic pancreatitis, but it also increases the rates of developing recurrent acute pancreatitis, pancreatic exocrine insufficiency and diabetes,^11^ and smoking makes it worse.^12^^,^^13^

The second part of the answer is highlighted by Wang et al.^2^ Using genetic epidemiology of a Han Chinese population, they confirmed that about half of all patients with idiopathic chronic pancreatitis have pathogenic mutations in the *CFTR, CTRC, PRSS1, SPINK1* genes, often in combination (see Zou,^14^ especially Supplementary Information pages 20-ff). Importantly, they also demonstrated a nearly 40% prevalence of the same mutations are in patients with both light-moderate alcohol consumption and heavy alcohol consumption—a highly significant statistical finding and a highly significant conceptual paradigm-shifting insight! Furthermore, the age of onset of pancreatitis in both groups of alcohol users, the rate progression, and the severity of complications were strongly dependent on the underlying genetic risks. Although some of these relationships have been previously reported,^15^, ^16^, ^17^ the careful genetic analysis^14^ and case stratification of alcohol users results in a better understanding of alcohol-associated pancreatitis, and extends findings in studies of mostly European ancestry studies to East Asian populations (ie these are universal principles).

The third part of the answer is in the future. Specifically—what else triggers acute pancreatitis in alcohol-drinking individuals and why is the immune response so aggressive in alcohol drinkers? We know that the most common etiology of acute pancreatitis is biliary. Since alcohol drinking and biliary disease risks are both common, we should now be asking whether a large fraction of alcohol-associated pancreatitis patients actually biliary acute pancreatitis patients that would benefit from a cholecystectomy or other measures. In addition, new insights into the mechanism of aggressive pancreatitis progression and greater complications in alcohol-associated chronic pancreatitis are also emerging. The *PRSS1-PRSS2* locus, thought to regulate *PRSS1* levels, acts as a major cofactor in alcohol-associated chronic pancreatitis.^18^ This high-risk haplotype may actually be altering T cell receptor beta repertoire and drive more the severe inflammation through immune dysregulation.^19^

Thus, the manuscript by Wang et al^2^ will help break the traditional thought that alcohol *causes* acute and chronic pancreatitis. Instead, we learn that alcohol (and smoking) make pancreatitis worse, but only in the context of other factors that we must be aware of and can prospectively address as we work to control and minimize the effects of this terrible condition.
